# Impact of PFAS exposure on lipid metabolic pathways: mechanisms and implications in carcinogenesis

**DOI:** 10.3389/ftox.2026.1768277

**Published:** 2026-03-11

**Authors:** Emily J. Ferguson, Josiane Weber Tessmann, Yekaterina Y. Zaytseva

**Affiliations:** 1 Department of Toxicology and Cancer Biology, University of Kentucky, Lexington, KY, United States; 2 Markey Cancer Center, University of Kentucky, Lexington, KY, United States

**Keywords:** cancer, carcinogenesis, forever chemicals, lipid metabolism, PFAS exposure

## Abstract

Per- and polyfluoroalkyl substances (PFAS) are a large class of synthetic chemicals widely used in industrial and consumer products owing to their unique surfactant properties. Their environmental persistence and bioaccumulative nature have resulted in widespread contamination of water, soil, and food sources, raising significant concerns for human health. Epidemiological and toxicological studies increasingly associate PFAS exposure with elevated risks of cancers, including liver, kidney, breast, and testicular cancers; however, the mechanisms underlying these associations remain incompletely understood. One emerging explanation is that PFAS disrupt lipid metabolism, a pathway frequently reprogrammed during cancer initiation and progression. PFAS share structural similarities with endogenous fatty acids and can bind to lipid transport proteins and/or activate lipid-sensitive nuclear receptors. Current evidence indicates that PFAS exposure is associated with increased blood lipid levels, as well as dysregulation of key transcription factors—such as peroxisome proliferator-activated receptors and sterol regulatory element-binding proteins—that can link PFAS exposure to tumor-promoting metabolic alterations. These disruptions can impair dietary fatty acid uptake and *de novo* lipid synthesis, leading to abnormal lipid accumulation, oxidative stress, and activation of pro-oncogenic signaling pathways. The purpose of this review is to synthesize current evidence on how PFAS exposure contributes to carcinogenesis through the disruption of lipid metabolism. By integrating findings from population studies, mechanistic research, and molecular insights, this review highlights lipid dysregulation as a critical connection between PFAS exposure and cancer biology and underscores the need for deeper investigation into this pathway.

## 1 Introduction

### 1.1 PFAS as environmental pollutants linked to the adverse effects on human health

Environmental contaminants have increasingly been implicated in the onset and progression of human diseases, largely through their capacity to disrupt biological homeostasis. Among these, per- and polyfluoroalkyl substances (PFAS) have emerged as persistent pollutants with broad bio-accumulative potential (National Academies of Sciences Engineering and Medicine, 2022). PFAS are a large, diverse class of synthetic chemicals, comprising over 15,000 distinct structures and have been extensively used in industry and consumer products worldwide since the 1940s (National Academies of Sciences Engineering and Medicine, 2022). Long carbon chain PFAS chemicals, i.e., perfluorooctanoic acid (PFOA) and perflurooctanecsulfonic acid (PFOS), also known as “legacy PFAS,” have been used in products for decades, and they are the most well-studied (Roth et al., 2021). Although legacy compounds such as PFOA and PFOS have been phased out in many regions, emerging replacements such as hexafluoropropylene oxide dimer acid (HFPO/GenX), and perfluorohexane sulfonic acid (PFHxS) have shown comparable, and in some cases greater, toxicity (Nian et al., 2020). Table 1 demonstrates structural differences of “legacy” and newer PFAS. During the production process and use of these products, PFAS substances can migrate into the soil, water, and air, contributing to widespread environmental contamination (Buck et al., 2011). Human exposure can be from contaminated drinking water, consumption of food that is contaminated through environmental exposure or packaging material (Buck et al., 2011). Other routes of exposure are occupational, such as via fire-fighting foam or chemicals used in manufacturing (Agency for Toxic Substances and Disease Registry, 2021).

**TABLE 1:** Structure of “legacy” and other emerging PFAS chemicals.

Compound	Structure	Reference
Legacy Compound: Perfluorooctanoic acid (PFOA)	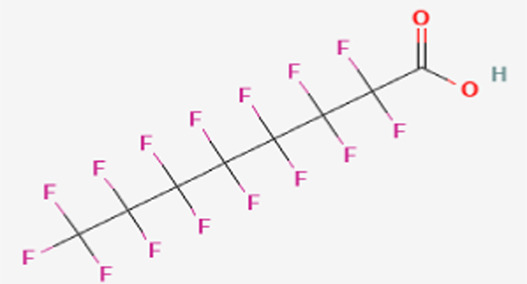	NCfBI (2026h)
Legacy Compound: Perfluorooctanesulfonic acid (PFOS)	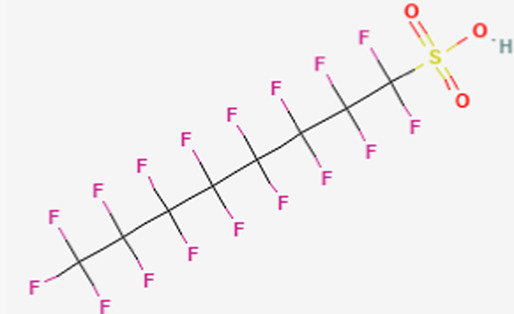	NCfBI (2026b)
Perfluorononanoic acid (PFNA)	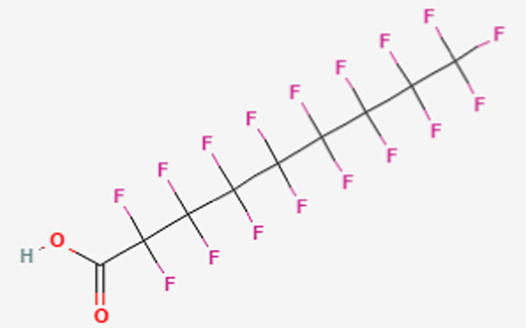	NCfBI (2026d)
Perfluorodecanoic acid (PFDA)	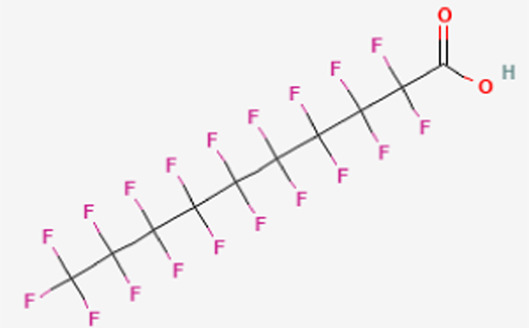	NCfBI (2026f)
Hexafluoropropylene oxide (HFPO)	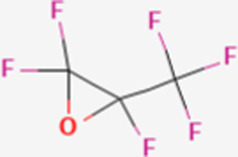	NCfBI (2026a)
Perfluoroheptanoic acid (PFHpA)	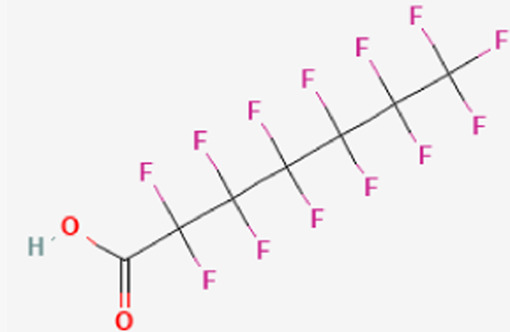	NCfBI (2026c)
Perfluorohexane sulfonic acid (PFHxS)	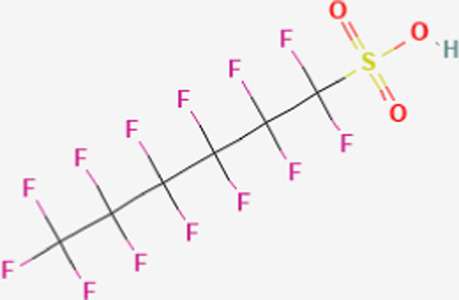	NCfBI (2026e)
Lithium bis [(trifluoromethyl)sulfonyl] azanide (HQ-115)	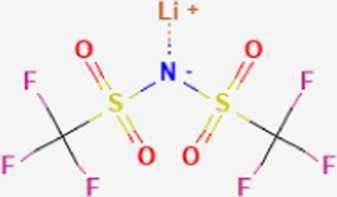	NCfBI (2026g)

Even though the effects of PFAS on human health are incompletely understood, multiple studies have linked contaminated drinking water to various adverse health outcomes including the increased risk of cancer. In the Mid-Ohio Valley, resident adults (age 18 years old or older) exposed to PFOA in drinking water from chemical plant emissions showed positive associations between cumulative serum PFOA concentrations and an increased risk of kidney and testicular cancer (Barry et al., 2013). The same cohort also demonstrated that increasing PFOA and PFOS levels were associated with elevated total cholesterol and low-density lipoprotein cholesterol (LDL-C) (Frisbee et al., 2010; Steenland et al., 2009). In Wilmington, North Carolina, residents exposed to both fluoro-ethers and legacy PFAS, PFOA, and PFOS through drinking water had higher total and non-HDL cholesterol, especially in older adults (Rosen et al., 2022). A Swedish cohort in Ronneby exposed to PFAS from firefighting foams showed elevated PFOS and PFHxS levels, which were associated with increased risks of polycystic ovarian syndrome, possible uterine leiomyoma, and moderately increased kidney cancer risk (Hammarstrand et al., 2021). Advanced machine learning utilizing the analysis association between PFAS and prostate-specific antigen metrics shows that elevated serum levels of perfluorodecanoic acid (PFDA), PFOA, PFOS, and perfluorononanoic acid (PFNA) are linked to prostate hyperplasia (Wang et al., 2024). These examples suggest a link between PFAS exposure, dysregulation of lipid metabolism, and carcinogenesis, as discussed in this review.

### 1.2 Population studies linking PFAS exposure to carcinogenesis

Growing epidemiological evidence shows that PFAS exposure is associated with an increased risk of various cancers (Liu et al., 2023). The National Academies’ 2022 report, “Guidance on PFAS Exposure, Testing, and Clinical Follow-Up,” states that PFAS exposure has been linked to a number of adverse health effects including certain cancers (National Academies of Sciences Engineering and Medicine, 2022). Human occupational and community exposure to PFAS has been associated with kidney, testis, breast, prostate, and liver cancer (National Academies of Sciences Engineering and Medicine, 2022).

A case-cohort study within the Dongfeng-Tongji cohort found that an increase of exposure of PFOA and perfluoroheptanoic acid (PFHpA) is associated with an elevated incident risk of breast cancer (Feng et al., 2022). The computational analysis of the four perfluorinated carboxylic acids (PFCAs) including PFOA, PFNA, PFDA, and PFHpA as a mixture, found that an increase in plasma concentrations of the four PFCAs was associated with a 19% increased incidence risk of breast cancer [HR (95%CI) = 1.19 (1.01, 1.41), *P* = 0.046]. PFOA, PFNA, and PFHpA accounted for 56%, 25%, and 19% of the positive effect, respectively (Feng et al., 2022). Another nested case-control study in the French E3N cohort found positively linear associations between PFOS serum concentrations and the risk of estrogen receptor and progesterone receptor positive breast cancer (Mancini et al., 2020). Low concentrations of PFOS and PFOA were associated with receptor-negative breast cancer (Mancini et al., 2020).

A study on the occupational effect of a mix of PFOA and PFOS on workers who had worked for at least 6 months in a factory producing these chemicals was done to identify their association with liver cancer and liver cirrhosis (Girardi and Merler, 2019). This study of 462 male employees showed an increased mortality due to liver cancer (SMR: 2.32; CI: 1.11-4.87) and liver cirrhosis (RR: 3.87; CI: 1.18-12.7) (Girardi and Merler, 2019). The PFOA serum levels of 120 workers in the selected time period (2000-2013) was very high (average of 4048 ng/mL) (Girardi and Merler, 2019). The mortality rates of these workers, with the addition of causes of liver cancer and liver cirrhosis, increased in association with the probability of PFAS exposure (Girardi and Merler, 2019).

PFAS exposure has also been linked to ulcerative colitis, which is recognized as a potential precursor for colorectal cancer (Durham et al., 2023). A positive link between PFOA exposure and ulcerative colitis has been reported in the occupational cohort of 3713 workers using lifetime serum cumulative dose (combining occupational and non-occupational exposure) as the exposure metric (Ste et al., 2015).

Kidney cancer has shown consistent associations with increasing PFAS levels. For example, a Swedish cohort studied the effect of kidney cancer outcomes with high PFAS exposure in drinking water (Li H. et al., 2022). This study showed a moderately increased risk of kidney cancer (HR 1.84; 95%CI 1.00-3.37) in subjects who lived in the contaminated water area where exposure was estimated to be highest (Li H. et al., 2022). These findings were consistent with previous studies that positively related PFOA exposure to kidney cancer (Barry et al., 2013; Ste et al., 2015; Vieira et al., 2013). In addition, a case-cohort study in the American Cancer Society’s CPS-II LifeLink cohort reported that serum PFOA concentrations were positively associated with renal cell carcinoma of the kidney among women (Winquist et al., 2023).

Epidemiological studies also link the PFAS exposure to testicular germ cell tumors (TGCTs) showing that several species of PFAS exhibiting pro-tumorigenic effects on TGCTs (Boyd et al., 2024; Purdue et al., 2023). The nested case-control study involved active-duty Air Force servicemen with serum from the Department of Defense Serum Repository show that elevated PFOS concentrations were positively associated with TGCT (Purdue et al., 2023).

Emerging evidence has also implicated PFAS in lung cancer progression. A study observed that serum PFOA levels in patients with advanced lung adenocarcinoma were significantly higher than those in patients with early-stage disease (Mei et al., 2024) suggesting a possible role in cancer progression or severity.

Associations have also been suggested between PFAS exposure and certain hematologic cancers. A case-cohort study suggested an association between PFHxS concentrations and chronic lymphocytic leukemia/small lymphocytic lymphoma among men (Winquist et al., 2023).

While population-based studies offer compelling evidence linking PFAS exposure to cancer risk (Table 2), they do not fully explain the biological mechanisms driving tumor development. A growing body of research suggests that the lipid-mimicking properties of PFAS may contribute to carcinogenesis by disrupting normal lipid metabolism.

**TABLE 2:** Summary of population studies linking PFAS to carcinogenesis.

Objective	Findings	References
To study the relationship between PFOA exposure and cancer in a worker cohort	Found a positive (but not statistically significant) association between PFOA exposure and prostate cancer; inverse association for bladder cancer	(Steenland et al., 2015)
To investigate mortality rates in men with high internal exposure to PFOA.	Observed increased mortality from testicular and kidney cancer, as well as other malignancies	(Girardi and Merler, 2019)
To examine serum PFAS concentrations and their relationship with breast cancer risk in the French E3N cohort	Reported positive associations between specific PFAS compounds (e.g., PFOS, PFOA) and breast cancer risk	(Mancini et al., 2020)
To evaluate cancer incidence in a Swedish population exposed to PFAS-contaminated drinking water	Found elevated incidence of kidney cancer and prostate cancer in highly exposed individuals	(Li et al., 2022a)
To provide guidance on PFAS exposure, testing, and clinical follow-up	Focused on exposure assessment, not specific cancer outcomes. However, it recognizes PFAS exposure as a risk factor for multiple cancers	(National Academies of Sciences Engineering and Medicine, 2022)
To assess the association between plasma PFAS exposure and breast cancer incidence in the Dongfeng-Tongji cohort	Found that higher plasma PFAS levels were associated with an increased risk of breast cancer	(Feng et al., 2022)
To investigate the association of serum concentrations of PFAS and testicular germ cell tumors among U.S Air Force servicemen	Higher PFOS serum concentrations were associated with an increased risk of testicular germ cell tumors among U.S. Air Force servicemen, while other PFAS showed weak, null, or inverse associations	(Purdue et al., 2023)
To assess associations between PFAS and selected cancers within the American Cancer Society’s CPS II LifeLink Cohort	Reported associations between PFAS exposure and kidney cancer, prostate cancer, chronic lymphocytic leukemia, small lymphocytic lymphoma, and testicular cancer	(Winquist et al., 2023)
To determine how PFOA triggers changes in the cellular mechanics of lung adenocarcinoma that leads to cancer metastasis	Patients with advanced lung adenocarcinoma had significantly higher PFOA serum levels than those patients with early-stage lung adenocarcinoma	(Mei et al., 2024)

### 1.3 Role of lipid metabolism in cancer

In their 2011 update to the Hallmarks of Cancer framework, Hanahan and Weinberg introduced the concept of metabolic reprogramming as one of the new hallmarks of cancer (Hanahan and Weinberg, 2011). This review focuses on lipid metabolism reprogramming and how its plays a large role in cancer growth and progression. There are two major sources of lipids: *de novo* lipid synthesis and dietary fatty acid uptake (Cheng et al., 2018; Cheng et al., 2022). Utilization of lipids allows cancer cells to sustain rapid growth, proliferation, and survival by providing essential building blocks for membranes, energy, and signaling molecules (Cheng et al., 2018; Cheng et al., 2022; Beloribi-Djefaflia et al., 2016; Bian et al., 2021; Broadfield et al., 2021; Chen and Huang, 2019; Currie et al., 2013; DeBerardinis et al., 2008).

The pathway of *de novo* lipid metabolism, also known as lipogenesis or lipid synthesis, involves the cellular production of lipids such as fatty acids, triglycerides, and cholesterol, from simpler, non-lipid precursors like glucose (Cheng et al., 2022). Key regulators in this process include Sterol Regulatory Element-Binding Proteins (SREBPs), which regulate expression of genes involved in lipid and cholesterol synthesis (Nian et al., 2020; Cheng et al., 2018). SREBPs are highly upregulated in several cancers and can promote tumor growth and survival (Nian et al., 2020; Cheng et al., 2018). In addition, fatty acid synthase (FASN), a key enzyme in *de novo* fatty acid synthesis, is shown to promote the initiation and progression of colorectal cancer by enhancing beta-catenin signaling and increasing stem-like properties of cancer cells (Kelson et al., 2024). The altered lipid metabolism also helps cancer cells to survive under metabolic stress due to nutrient deficiency and hypoxia within the tumor microenvironment (Fu et al., 2021; Molendijk et al., 2020). This dysregulation can also modulate ferroptotic-mediated cells in circulating tumor cells during the metastatic process (Fu et al., 2021). This reduces the oxidative stress of the cancer cells and increases their survival during migration through the blood (Fu et al., 2021).

Dietary lipid metabolism refers to the uptake and processing of pre-formed lipids from external sources, primarily through the diet (Maerten et al., 2024). This pathway involves the absorption of lipids from the digestive system, their transport into the bloodstream, and uptake by the cells through specific transporters such as Fatty Acid Binding Protein 4 (FABP4) and Fatty Acid Translocase, also known as CD36 (Birchfield et al., 2025; Wan et al., 2012). Upregulation of fatty acid transporters is commonly associated with carcinogenesis and tumor aggressiveness (Pascual et al., 2017; Zhao et al., 2017; Acharya et al., 2023). Dividing tumor cells require significant amounts of fatty acids for membrane synthesis and cholesterol to maintain their membrane integrity and function (Cheng et al., 2018; Currie et al., 2013). Cancer cells often store excess cholesterol as cholesteryl esters (CEs) and fatty acids as triglycerides (TGs) within intracellular lipid droplets (LDs) (Cheng et al., 2018; Geng et al., 2023; Gupta et al., 2024). These LDs act as critical reservoirs for maintaining energy homeostasis in cancer cells (Geng et al., 2023; Geng and Guo, 2024). The mobilization of stored lipids, often via autophagy (hippophagy), is a pro-survival mechanism that enables cancer cells to maintain growth and resist various cancer therapies (Geng and Guo, 2024).

PFAS chemicals, due to their structural similarity to endogenous fatty acids and their ability to bind to lipid transport proteins and activate lipid-sensitive nuclear receptors, are increasingly recognized as metabolic disruptors (Hammarstrand et al., 2021; Birchfield et al., 2025; Vanden Heuvel et al., 2006; Wolf et al., 2008). These compounds can dysregulate both *de novo* lipid synthesis and fatty acid uptake, thus contributing to both cancer initiation and progression.

### 1.4 Link between structural properties of PFAS and lipid metabolism

PFAS are referred to as “forever chemicals” due to their high thermal and chemical stability. They exhibit minimal biodegradability and can bioaccumulate in the body over time (Agency for Toxic Substances and Disease Registry, 2021). Structurally, PFAS consist of a perfluoroalkyl chain and a functional head group (such as carboxylic acid, sulfonate, alcohol, phosphate, amino) attached to a nonfluorinated hydrocarbon (Agency for Toxic Substances and Disease Registry, 2021). Variations in structure, including chain length and branching, determine their behavior, classification, and nomenclature (Agency for Toxic Substances and Disease Registry, 2021). The exceptional stability of PFAS arises from the unique properties of the carbon-fluorine (C–F) bond (Agency for Toxic Substances and Disease Registry, 2021). Their lipid-like properties allow PFAS to act as molecular mimics, activating lipid-sensitive nuclear receptors such as peroxisome proliferator-activated receptors (PPARs), which can dysregulate lipid metabolism through altered gene expression pathways (Buck et al., 2011). Notably, PFOA and PFOS are classified as “perfluorinated fatty acid analogs” and their structural resemblance to natural fatty acids underlies their biological activity (Vanden Heuvel et al., 2006).

Studies on PFAS ability to bind to proteins like FABP4 show that “hydrophobic interactions within the binding cavity” are enhanced by these PFAS structures, which explains their strong binding affinities (Birchfield et al., 2025). The chain length and headgroups of PFAS are critical factors in these interactions, similar to how different fatty acid structures interact with proteins (Wolf et al., 2008).

Liver fatty acid binding proteins (L-FABP) are crucial proteins that regulate the transport of PFAS (Yang et al., 2020). L-FABPs deliver PFAS to PPARs and thus regulate the signaling pathways of PPARs (Yang et al., 2020). FABP protein can form a complex with CD36 on the cytoplasmic side of the cell membrane, promoting the uptake of PFAS in the cell (Sheng et al., 2018). Notably, while short-chain (n < 8) PFAS structurally resemble endogenous fatty acids, studies have shown that even long-chain PFAS (n > 8) can still bind to L-FABP and be transported into the cell nucleus (Sheng et al., 2018).

The strong protein-binding affinity of PFAS and their capacity to dysregulate lipid metabolism have raised significant concerns regarding long-term health outcomes in exposed populations.

**FIGURE 1:**
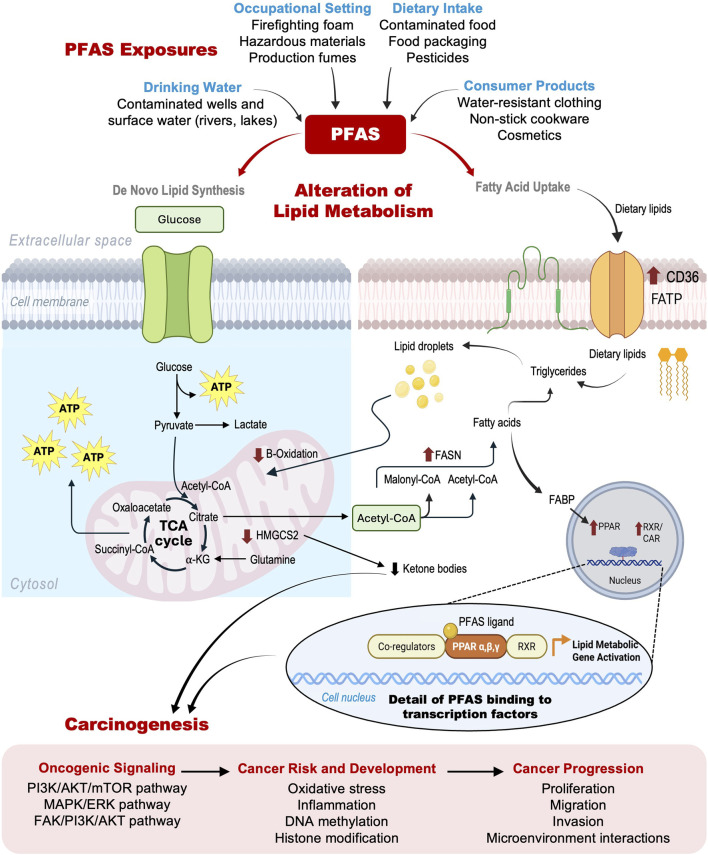
Schematic overview illustrating the link between PFAS exposure, lipid metabolism dysregulation, and cancer progression. Top: Primary routes of PFAS exposure (from most to least abundant: drinking water, occupational settings, dietary intake, and consumer products) along with representative sources for each route. Middle: PFAS-induced disruption of lipid metabolism, highlighting interference with *de novo* lipid synthesis and fatty acid uptake pathways; red arrows indicate PFAS effects on specific enzymes and pathways. Detail panel depicts PFAS binding to transcription factors such as PPARs, leading to altered expression of lipid metabolic genes. Bottom: Downstream consequences of PFAS exposure, illustrating the progression from oncogenic signaling activation to increased cancer risk, tumor development, and eventual metastasis and invasion.

However, several emerging PFAS substitutes, including GenX and HQ-115, possess chemical structures that differ substantially from those of legacy PFAS (Figure 1) and long-chain fatty acids, yet they still disrupt lipid metabolism-associated signaling pathways (Shi et al., 2023; Boyd et al., 2025; Dunn et al., 2024; Sands et al., 2024; Zhang et al., 2025). These observations highlight critical knowledge gaps regarding the molecular mechanisms by which these newer compounds reprogram lipid metabolic networks and contribute to carcinogenesis, underscoring the urgent need for systematic mechanistic and translational studies.

## 2 The mechanisms linking PFAS, lipid metabolism and carcinogenesis

PFAS are generally considered non-mutagenic, meaning they do not directly induce DNA mutations (Buck et al., 2011). However, increasing evidence indicates that PFAS contribute to carcinogenesis through DNA damage, epigenetic modifications, and alterations of signaling and metabolic pathways, thereby creating a pro-carcinogenic cellular environment (Winquist et al., 2023). These molecular disruptions are closely linked to dysregulated lipid metabolism, a hallmark of cancer metabolic reprogramming. Multiple studies demonstrate that PFAS interfere with normal lipid homeostasis, leading to hepatotoxicity, dyslipidemia, and aberrant lipid uptake, synthesis, and storage across various cell types which are all processes associated with elevated cancer risk (Sadrabadi et al., 2024; Haug et al., 2023; India-Aldana et al., 2023; Boyd et al., 2022; Kirkwood-Donelson et al., 2024; Zaytseva, 2021; Das et al., 2017; Wang et al., 2013; Yu et al., 2023).

### 2.1 Lipid accumulation and steatosis

Experimental evidence shows that PFAS exposure elicits dose-dependent metabolic changes (Das et al., 2017; Louisse et al., 2020). In hepatocyte models, a PFAS mixture containing 47.8% PFOS, 28.7% PFOA, 12.1% PFHxS, 6.3% PFDA, and 5.1% PFNA induced broad metabolic shifts, notably affecting the metabolism of lipids, steroids, bile acids, amino acids, and carbohydrates (Boyd et al., 2025). PFAS exposure led to dysregulation of pathways associated with oxidative stress, function of mitochondria, cytoskeleton, and lipid-rich membranes (Boyd et al., 2025). In human HepaRG liver cells show that PFOA, PFOS, and PFNA increased intracellular triglyceride levels (Louisse et al., 2020). PFOS exposure further induced lipogenic gene expression, and excessive fatty acid and triglyceride accumulation, promoting hepatic steatosis and fibrosis (Wan et al., 2012; Marques et al., 2020).

These steatotic responses align with findings that PFOA- and PFOS-mediated lipid accumulation promotes non-alcoholic fatty liver disease (NAFLD), a risk factor for liver cancer (Gou et al., 2024). Mechanistically, PFAS exposure downregulated Nudix hydrolase 7, reducing Ace-CoA hydrolase activity and driving lipid accumulation in hepatocytes (Gou et al., 2024).

Population-level evidence supports these findings. Analysis of the National Health and Nutrition Examination Survey (NHANES) data revealed a significant association between PFAS exposure and hepatic steatosis/NAFLD (Wu et al., 2023). Transcriptomic and bioinformatic analyses identified transcriptional upregulation of hepatic acyl-CoA oxidase 1 (ACOX1) in the PPARα-regulated peroxisomal β-oxidation pathway as a key event in PFAS-perturbed lipid metabolism in humans, mice, and rats (Yang W. et al., 2023). This ACOX1-mediated oxidative stress can contribute to mitochondrial compromise and lipid accumulation (Yang W. et al., 2023).

### 2.2 Disruption of de novo lipid metabolism

Beyond lipid accumulation, PFAS alter *de novo* lipid biosynthesis by modulating fatty acid and cholesterol metabolism (Yang W. et al., 2023; Cao et al., 2022; Hari et al., 2024; Sim et al., 2023). In HepaRG cells, PFOS and PFOA elevated multiple lipid species, including ceramide, diacylglycerol, N-acylethanolamine, phosphatidylcholine, and triglycerides (Kashobwe et al., 2024a). In mouse liver, PFOS exposure causes hepatic steatosis by significantly increasing TG levels, expression of ATP-citrate lyase, and FASN (Lee et al., 2023). Integrated transcriptomic and lipidomic analyses of epithelial cells chronically exposed to PFOA and PFOS revealed enhanced lipid biosynthesis via the SREBP axis and compound-specific alterations in membrane lipids (Paddayuman et al., 2025).

PFAS-induced lipid dysregulation also extends to extrahepatic tissues. Low-dose PFOA exposure enhanced fatty acid metabolism and steroidogenic processes in Leydig cells, stimulating steroid hormone synthesis (Huang et al., 2022). Similarly, PFOS localized within mouse testicular tissue and increased levels of fatty acid metabolites such as palmitic acid, oleic acid, stearic acid, and cholesterol (Boyd et al., 2024) In humans, PFOS, PFOA, and PFDA were positively associated with cholesterol, lipoprotein subfractions, apolipoproteins, and composite fatty acid and phospholipid profiles (Haug et al., 2023), indicating systemic alterations in lipid metabolism.

Consistent with these findings, our group reported that PFOS exposure through drinking water downregulated mitochondrial hydroxy- 3methylglutaryl CoA synthase 2 (HMGCS2), a rate-limiting enzyme in ketogenesis (Tessmann et al., 2024). Because HMGCS2 governs ketone body production, its inhibition may alter tumor metabolism and the tumor microenvironment (Wei et al., 2022). PFOS exposure concurrently upregulated β-catenin, c-MYC, mTOR, and FASN which are key regulators linked to colorectal cancer (Tessmann et al., 2024).

### 2.3 Lipid transport and uptake dysregulation

In addition to modifying lipid synthesis, PFAS disrupt lipid transport and uptake mechanisms. PFOS increased gene expression of CD36 and lipoprotein lipase, enhancing triglyceride breakdown and cellular lipid absorption (Wan et al., 2012). Conversely, certain PFAS downregulated lipid transport genes including Apoa1, Apoa5, and Pltp (Hari et al., 2024), suggesting a complex and potentially varied impact of PFAS exposure on lipid transport mechanisms depending on the specific PFAS and environment.

PFAS also interact directly with lipid-binding proteins. FABP4, predominantly expressed in adipocytes, binds a broad range of PFAS (Birchfield et al., 2025). Because FABP4 regulates fatty acid transport, adipogenesis, insulin sensitivity, and cancer-related metabolic processes (Prentice et al., 2021), PFAS–FABP4 interactions may directly perturb lipid signaling and promote oncogenic metabolic phenotype (Jia et al., 2024).

### 2.4 Bile acid signaling and enterohepatic circulation

PFAS further affect bile acid homeostasis, an essential regulator of cholesterol and lipid metabolism (Fleishman and Kumar, 2024). Bile acid dysregulation has been implicated in tumorigenesis, as bile acid transporters influence several stages of cancer progression (Bintee et al., 2025). In male mouse ileum, PFAS exposure significantly upregulated the apical sodium-dependent bile acid transporter (ASBT), increasing bile acid reabsorption and altering enterohepatic circulation (Roth et al., 2024). ASBT is a crucial transporter responsible for actively absorbing approximately 95% of bile acids from the intestinal lumen back into ileal enterocytes, playing a central role in enterohepatic circulation (Roth et al., 2024). Transcriptomic analysis confirmed PFAS-induced alterations in fatty acid metabolism pathways (Roth et al., 2024). These findings are consistent with human studies demonstrating that ASBT contributes to PFAS reabsorption and accumulation through enterohepatic recirculation (Cao et al., 2022; Zhao et al., 2015).

### 2.5 Peroxisome proliferator-activated receptors

PPARs are a family of nuclear receptors, which include three main subtypes, PPARα, PPARγ, and PPARδ, that act as ligand-activated transcription factors (Berger and Moller, 2002). They play a critical role as lipid sensors and regulators of lipid metabolism by controlling gene expression (Berger and Moller, 2002). In normal, non-cancerous tissues, PFAS bind and activate PPARα, leading to its transactivation and changes in the expression of genes in lipid and carbohydrate metabolism, primarily in the liver (Vanden Heuvel et al., 2006). Heuval et al. (2006) reported that PFOA strongly activates PPARα in human, rat, and mouse tissues, compared to other nuclear receptors including PPARβ, PPARγ, Liver X Receptor Beta (LXRβ), and Retinoid X Receptor Alpha (RXRα) (Vanden Heuvel et al., 2006). Similarly, Sadrabadi et al. (2024) demonstrated that PFAS mixtures induce triglyceride accumulation and upregulate PPARα target genes related to lipid and cholesterol metabolism in differentiated HepaRG cells (Sadrabadi et al., 2024).

Notably, the PFAS-mediated activation of mouse PPARα is generally higher compared to that of human PPARα (Wolf et al., 2008). While PFOA and PFOS act mainly through PPARα transactivation in rodents, non-rodent species (including humans) exhibit a reduced response to these effects (Bjork et al., 2011). Although most PFAS are PPARα activators, some, like perfluoro-butane sulfonic acid (PFBS), might not activate human PPARα (Behr et al., 2020). Conversely, understudied PFAS such as PFOSA, 6:2 FTOH, and 8:2 FTSA can downregulate PPARα gene expression, indicating mechanistic diversity among PFAS compounds (Kashobwe et al., 2024b).

Activation of PPARα by PFAS has been implicated in NAFLD and liver toxicity, which may increase the risk of liver carcinogenesis (Yang W. et al., 2023; Li et al., 2019). The activation of PPARα and related pathways by PFAS may also have different consequences in malignant cells, where metabolic processes are reprogrammed to support rapid proliferation and survival (Yang W. et al., 2023). In HepG2 hepatocellular carcinoma cells, PFOA, PFOS, and several alternative PFAS (except PFBS) activate human PPARα, while Perfluoro-2-methyl-3-oxahexanoic acid (PMOH) and PFOA also act as weak agonists of PPARγ (Behr et al., 2020). GenX exposure, even at environmental concentrations, disrupts hepatic lipid metabolism via the PPARα signaling pathway affecting fatty acid transport, synthesis, and oxidation in male C57BL/6 mice (Shi et al., 2023).

In a recent study, PFOS and HQ-115 altered metabolites involved in steroid biosynthesis and lipid metabolism in testicular germ cell tumor (TGCT) cells, consistent with the ability of PFAS to mimic fatty acid-based ligands that regulate lipid metabolism and with their proposed role as endocrine disruptors (Boyd et al., 2024). Moreover, the follow-up study showed that PFOS, GenX, and HQ-115 exposure disrupted PPAR signaling, with the most prominent effect being antagonistic activity toward PPARγ (Boyd et al., 2025). These findings suggest that PPARγ may be particularly sensitive to PFAS exposure in TGCT patients. However, additional *in vivo* and human-based studies are required to validate these observations and determine their clinical relevance.

### 2.6 Sterol regulatory element-binding proteins

SREBP1 and SREBP2 are key regulators of lipogenesis and cholesterol synthesis and have been linked to PFAS-induced metabolic effects (Louisse et al., 2020; Yan et al., 2015). Low concentrations of PFOA exposure enhances survival of cancer liver cells by upregulating expression of SREBP1 and its downstream targets, FASN and ACC (Qi et al., 2023). In mice, PFOA and GenX induce hepatic expression of SREBP-1 target genes involved in fatty acid synthesis (Attema et al., 2022). Interestingly, this effect is absent in PPARα^−/−^ mice, suggesting that these effects are PPARα-dependent (Attema et al., 2022). Another study found that 28-day PFOA exposure enhanced both PPARα and SREBP transcriptional activity, stimulating SREBP maturation and transcriptional activation (Yan et al., 2015).

### 2.7 Constitutive androstane receptor (CAR) and pregnane X receptor (PXR)

Although PFAS effects are primarily linked to PPARα, they can also act through other nuclear receptors such as CAR and PXR, which regulate energy metabolism and hepatic lipogenesis (Bjork et al., 2011). In the absence of PPARα, PXR and CAR becomes more prominent in mediating the effects of PFOA (Bjork et al., 2011; Rosen et al., 2017). In Sprague-Dawley rats, PFOS-induced hepatic hypertrophy and adenomas were associated with activation of PPARα, CAR, and PXR (Elcombe et al., 2012). Conversely, studies in HEK293T and HepG2 cells found no activation of CAR or PXR by PFAS, suggesting compound- or model-specific variability (Behr et al., 2020). A mixture of PFOS and PFOA also exerted a synergistic effect on breast cancer cell proliferation mediated through PXR activation (Pierozan et al., 2020).

### 2.8 Hepatocyte nuclear factor 4 alpha

HNF4α is a nuclear receptor expressed in the liver, gut, kidney, and pancreas that regulates genes involved in metabolism and is upregulated in multiple malignancies, including hepatocellular, colorectal, and pancreatic cancers (Sang et al., 2022). Molecular docking and dynamics simulations identify HNF4α as a potential target in PFOS- and PFOA-induced hepatic steatosis (Li et al., 2024). In human hepatocytes, PFOA and PFOS promote dedifferentiation and proliferation by downregulating positive HNF4α targets (e.g., CYP7A1) and inducing negative, pro-mitogenic targets (e.g., CCND1) (Beggs et al., 2016). In HepG2 cells, PFOA decreases HNF4α and its downstream regulator HNF1α, reducing expression of fatty acid oxidation genes (e.g., ACOX1) and promoting lipid accumulation (Scharmach et al., 2012). Together, these findings indicate that PFOS and PFOA can promote pathological liver changes and tumorigenesis through HNF4α-dependent mechanisms.

### 2.9 Pro-oncogenic signaling pathways

PFAS exposure can regulate cancer-associated signaling pathways, contributing to cancer initiation and progression (Zheng et al., 2024). In breast cancer cells, PFAS compounds promote cellular migration and invasion through the activation of oncogenic MAPK/ERK and PI3K/Akt signaling pathways via estrogen receptors, including ERα and G protein-coupled estrogen receptor (Liu et al., 2023; Hanahan and Weinberg, 2011; Gupta et al., 2024). Similarly, PFOA has been found to activate the intracellular FAK-PI3K-Akt pathway in lung cancer cells, further supporting a role for PFAS in enhancing pro-tumorigenic signaling cascades (Mei et al., 2024). In colorectal cancer, PFOS exposure leads to the upregulation of proteins such as β-catenin, c-MYC, and mTOR (Boyd et al., 2024). The Wnt/β-catenin pathway, crucial for prenatal liver development, is affected by early PFOS exposure, suggesting a potential developmental window of susceptibility to PFAS-induced carcinogenesis (Tessmann et al., 2024; Lai et al., 2017). Recent multi-omics and computational analyses have identified molecular targets linking PFAS exposure to carcinogenic metabolic signaling. In hepatocellular carcinoma, PFAS were found to modulate genes involved in lipid, glucose, and drug metabolism, including APOA1, ESR1, IGF1, PPARGC1A, SERPINE1, and PON1 (Hong et al., 2025). Molecular docking simulations demonstrated strong binding affinities between PFAS compounds and these proteins, supporting their potential roles in PFAS-induced liver carcinogenesis (Hong et al., 2025).

### 2.10 Oxidative stress and inflammation

Oxidative stress, a result of an imbalance between the production of reactive oxygen species (ROS) and the antioxidant system, is a common event in cancer pathophysiology (Li S. Q. et al., 2025). This imbalance can alter cellular lipid metabolism by promoting fatty acid synthesis and altering lipid utilization, which in turn supports cancer cell proliferation and survival (Li S. Q. et al., 2025). PFAS exposure has been shown to induce oxidative stress in multiple models (Gou et al., 2024; Qi et al., 2023; Li S. Q. et al., 2025; Yang Z. Y. et al., 2023; Alijagic et al., 2024; Wang et al., 2017; Hocevar et al., 2020; Kamendulis et al., 2014; Lee et al., 2015; Lin et al., 2020). PFOA exposure increases lipid peroxidation (8-iso-PGF_2_α) and induces antioxidant response genes (Sod1, Sod2, Gpx2, Nqo1) in mouse pancreas (Kamendulis et al., 2014). In pancreatic acinar cells, PFOA treatment activates the unfolded protein response (UPR), contributing to oxidative stress (Qi et al., 2023; Hocevar et al., 2020). In liver cells, PFAS exposure, including PFOA, heptafluorobutyric acid (HFBA), and perfluorotetradecanoic acid (PFTA), elevated ROS levels (Qi et al., 2023).

PFAS-mediated lipid accumulation can further induce oxidative stress and inflammation (Alijagic et al., 2024; Li X. et al., 2025). In zebrafish embryos, PFOA and PFOS disrupt lipid metabolism and reduce DHA-containing glycerophospholipids while activating proinflammatory signaling (Yang Z. Y. et al., 2023). In Taiwanese adults, serum PFOA and PFOS levels correlate positively with LDL-C, HDL-C, triglycerides, and urinary oxidative stress biomarkers (Lin et al., 2020).

In HepaRG and HepG2 cells, PFOA, HFBA, and PFTA increase expression of inflammatory markers TNFα and IL6 (Qi et al., 2023). Similarly, PFOA-induced changes in the fetal liver of CD-1 mice include genes such as the suppression of IL6r (gp80) and Stat3 via IL6 (Rosen et al., 2007). PFOS induced inflammatory gene expression occurs in both wild-type and PPARα-null mice, with stronger effects in the latter, indicating a PPARα role in PFAS-mediated inflammation (Rosen et al., 2010). Additional PPARα-independent effects may involve PPARγ and PPARβ/δ activation (Rosen et al., 2010). PFAS can also stimulate inflammation in immune cells by activating the AIM2 inflammasome, inducing IL-1β release and pyroptosis in macrophages (Wang et al., 2021), and dysregulating inflammatory interleukins and lipid-related genes in lymphocytes (Li et al., 2020).

### 2.11 Epigenetic perturbations: DNA methylation and histone modification

PFAS exert both direct epigenetic effects and indirect influence through lipid metabolism disruption, creating a dual mechanism that increases disease and cancer risk (Imir et al., 2021). PFOS and PFOA induce global DNA hypomethylation and histone modification changes associated with tumorigenesis (Boyd et al., 2024; Boyd et al., 2022; Pierozan et al., 2020; Wen et al., 2020; Ouidir et al., 2020; Pang et al., 2024). Global DNA methylation of HepG2 cells were analyzed after different levels of exposure to PFOA and GenX (Wen et al., 2020). From this study, there was an observed dose dependent reduction of methylation caused by PFOA (Wen et al., 2020). While the role that PFOA played on each TET was different, this difference suggested that epigenetic changes caused by PFOA can increase lipid anabolism and storage through altering cell homeostasis (Wen et al., 2020). PFOS exposure was also shown to induce gene expression alterations associated with the H3K27me3 polycomb pathway in normal mouse testes, suggesting its role in promoting pro-tumorigenic effects in testicular germ cell tumors (Boyd et al., 2024). Particularly when combined with dietary factors like a high-fat diet, PFAS exposure leads to both metabolic alterations and epigenomic reprogramming that increase tumorigenic risk in prostate cancer xenografts, suggests a complex interplay where both mechanisms contribute to the overall pro-carcinogenic effect (Imir et al., 2021).

The possible mechanisms linking PFAS exposure, lipid metabolism and carcinogenesis are summarized in Figure 1.

## 3 Potential intervention strategies to mitigate PFAS-Induced dysregulation of lipid metabolism to reduce cancer risk

Only a few studies suggest strategies to mitigate the toxic effects of PFAS chemicals on their dysregulation of lipid metabolism. A study by Li, R., et al. (2022) demonstrated that vitamin C supplementation reduced signs of PFOA-induced liver damage in mice, including increased total cholesterol and triglyceride levels, elevated liver damage markers (aspartate and alanine transaminase), and liver enlargement (Li R. et al., 2022). Another *in vitro* study showed that PFOA treatment of mouse pancreatic acinar cells resulted in endoplasmic reticulum stress and activation of the UPR pathway (Hocevar et al., 2020). PFOA-stimulated activation of the UPR was blocked by pretreatment with specific PERK and IRE1alpha inhibitors and the chemical chaperone 4-phenyl butyrate (Hocevar et al., 2020). This suggests potential chemical interventions targeting these pathways. A study by Yang et al. (2023) used an integrated approach including *in vitro* assays with mouse hepatocytes (Yang W. et al., 2023). They observed that mitochondrial compromise and lipid accumulation in PFOA/PFOS-exposed mouse hepatocytes could be mitigated by co-treatment with ACOX1 inhibitor and mitochondria ROS scavenger (Yang W. et al., 2023). This observation could lead to potential molecular targets for intervention in PFAS-induced liver metabolic disorders.

Several studies tested potential remedies but found them ineffective or with limited effects. For example, one study tested whether dietary choline supplementation attenuates PFOS-induced hepatic steatosis in male Sprague Dawley rats, but this intervention was ineffective in this experiment (Bagley et al., 2017). Another study examined the effects of PFOS exposure on intestinal tissues in mice, and tested whether diets containing soluble fibers inulin and pectin could reverse the adverse effects of PFOS (Deng et al., 2022). Transcriptomic analysis revealed that the number of differentially expressed genes associated with hepatic hyperlipidemia and lipid metabolism pathways (e.g., PPAR signaling, fatty acid degradation) was lower in inulin- and pectin-fed mice compared to control diet-fed mice after PFOS exposure (Deng et al., 2022). This suggests that the soluble fiber diets partially-rescued the lipid and metabolite disturbances caused by PFOS (Deng et al., 2022). While the high fiber diets improved lipid metabolism in the liver, this diet did not restore expression of ketogenic enzyme HMGCS2 in mouse intestinal tissues (Tessmann et al., 2024), suggesting the limitation of this strategy in mitigating the harmful effect of PFOS in intestine. Furthermore, supplementation with DHA was ineffective in recovering the lipidomic dysregulations and protecting zebrafish embryos from developmental toxicity induced by PFAS (Yang Z. Y. et al., 2023).

These mixed outcomes highlight the complexity of PFAS-induced metabolic disturbances and underscore the urgent need for more targeted and effective therapeutic approaches. Further research is critical to better understand the mechanistic underpinnings of PFAS-induced lipid metabolism disruption and to develop reliable strategies for prevention and intervention. Table 3 provides the summary of potential intervention strategies to mitigate the effect of PFAS on lipid metabolism.

**TABLE 3:** Summary of possible mitigation strategies against PFAS-induced lipid metabolic disruptions.

Objective	Finding(s)	References
Test whether choline reduces PFOS-induced hepatic steatosis in male Sprague Dawley rats	Ineffective at attenuating PFOS-induced hepatic steatosis	Bagley et al. (2017)
Determine if PFOA-induced ER stress and UPR activation can be chemically blocked in mouse pancreatic acinar cells	Activation of the UPR was blocked by PERK inhibitors, IRE1ɑ inhibitors, and chemical chaperone 4-phenyl butyrate, suggesting these as potential intervention targets	Hocevar et al. (2020)
Test whether vitamin C supplementation reduces PFOA-induced liver toxicity in mice	Vitamin C reduced PFOA-induced increases in total cholesterol, triglycerides, AST/ALT liver damage markers, liver enlargement, and liver enlargement-associated pathology	Li et al. (2022b)
Test whether soluble fibers reverse PFOS-induced lipid metabolic disruption	Lower number of lipid metabolism-related DEGs vs. control diet; liver lipid and metabolite disturbances were partially rescued via PPAR signaling and fatty acid degradation pathways, but did not restore intestinal ketogenic enzyme HMGCS2, showing limitations for intestinal protection	Deng et al. (2022)
Determine if DHA protects against PFAS-induced developmental lipidomic toxicity	Ineffective at restoring lipidomic disruptions or preventing developmental toxicity in zebrafish embryos	Yang et al. (2023b)
Assess mitigation of mitochondrial dysfunction and lipid accumulation in PFOA/PFOS-exposed mouse hepatocytes	Co-treatment with ACOX1 inhibitor and mitochondrial ROS scavenger reduced mitochondrial compromise and lipid accumulation, identifying possible molecular targets	Yang et al. (2023a)

## 4 Discussion

PFASs have been in industrial use since the 1940s; however, the first studies showing the presence of these compounds in the blood of exposed workers came out in the 1970s and studies on PFAS in the blood of the general population came out in the 1990s (National Academies of Sciences Engineering and Medicine, 2022; Grandjean and Clapp, 2015; Mueller and Schlosser, 2020). Since this time, multiple research studies have reported the harmful effects of these compounds on human health including cancer. The influence of PFAS chemicals on cancer biology, through the alteration of metabolic pathways is of growing concern in literature (Maerten et al., 2024; Hong et al., 2025; Guo et al., 2022; Boyd et al., 2022; Hu et al., 2022). There is growing interest in how PFAS might influence these metabolic pathways (Gou et al., 2024). While PFAS are well-documented for their role in lipid dysregulation in non-cancer models, especially in the liver, there is a recognized need for further understanding of their specific contribution to lipid metabolism in the context of cancer (Maerten et al., 2024; Hong et al., 2025; Alijagic et al., 2024; Imir et al., 2021).

There is significant impact of PFAS chemicals on lipid metabolism and their potential role in carcinogenesis (Boyd et al., 2022). PFAS, characterized by their strong chemical stability and lipid-mimicking properties, disrupt cellular lipid homeostasis by altering both *de novo* lipid synthesis and dietary lipid uptake pathways (Gou et al., 2024; Deng et al., 2022). These disruptions are mediated through the activation and dysregulation of key transcription factors and nuclear receptors, specifically PPARα, SREBPs, CAR, and PXR, which collectively drive abnormal lipid accumulation metabolic reprogramming, and oncogenic signaling (Nian et al., 2020; Maerten et al., 2024), PFAS-induced lipid metabolic changes also contribute to oxidative stress, inflammation, and epigenetic modifications such as DNA methylation and histone changes, which further promote tumorigenesis and cancer progression (Boyd et al., 2022). Epidemiological evidence supports associations between PFAS exposure and increased risk of various cancers, notably liver (Hong et al., 2025), breast (Liu et al., 2023), kidney (Behr et al., 2018), and testicular (Imir et al., 2021) cancers, underscoring the public health relevance of these findings.

However, our current understanding of mechanism behind PFAS exposure is limited by several factors. Many mechanistic studies rely on *in vitro* or animal models that may not fully represent human exposure (Roth et al., 2024; Bjork et al., 2011). For example, the toxicological effects of PFAS, such as PFOA and PFOS, are often linked to the activation of the PPARα (Wolf et al., 2008; Bjork et al., 2011). However, mouse PPARα generally appears more sensitive to PFAAs than human PPARα (Wolf et al., 2008). This difference in receptor activity contributes to the fact that while PFAS exposure causes a substantial shift toward fatty acid oxidation and hepatic triglyceride accumulation in rat liver cells, the observed changes in primary human liver cells are more subtle (Bjork et al., 2011; Rosen et al., 2013). Furthermore, the proliferative hepatic response seen in rats due to the activation of PPARα and CAR/PXR by PFAS is not expected to be relevant in humans (Elcombe et al., 2012). PFAS elimination kinetics vary widely by species (ITRC, 2023). The biological half-life of PFOS, for instance, is estimated to be years in humans (i.e., 3.4 years) but only months (i.e., 1–2 months) in rodents (Deng et al., 2022). Differences in how PFAS are handled by specific transporters are also observed. For example, the ASBT in human transports PFOS, but rat ASBT does not transport PFOS, PFHxS, or PFBS (Zhao et al., 2015). Also, studies using immortalized cell lines (e.g., HepG2, a male cell line, or HepaRG, a female cell line) or primary hepatocytes sometimes fail to fully recapitulate the complex metabolic pathways observed *in vivo* (Alijagic et al., 2024; Rosen et al., 2013). For example, several genes typically regulated by PPARα agonists *in vivo* in mice were not altered in cultured mouse hepatocytes (Rosen et al., 2013).

Importantly, research models often use doses or exposure scenarios that are too high, not representing the chronic low-dose exposures that are typical of environmental contamination (Buck et al., 2011; Rosen et al., 2007). Many *in vitro* effects observed on key molecular targets, such as nuclear receptors, occur only at high concentrations, often exceeding 10 μM (Sadrabadi et al., 2024; Behr et al., 2020; Behr et al., 2018). These concentrations are several magnitudes above the typical average human blood concentration, which is often in the nanomolar range or below (Behr et al., 2020; Behr et al., 2018). Even in animal studies intended to be relevant, the resulting circulating PFAS levels in mice can still be substantially elevated, sometimes up to approximately 256-fold higher than those reported in highly exposed human occupational cohorts (Roth et al., 2024; Deng et al., 2022). While low, physiologically relevant concentrations of PFAS have been shown to induce effects in human liver cell models like activating the unfolded protein response pathway and increasing steatosis, the majority of strong findings derived from *in vitro* or animal models rely on concentrations significantly higher than typical environmental exposure levels (Shi et al., 2023; Qi et al., 2023).

Moreover, variability in PFAS species, chain lengths, and exposure contexts complicates the generalization of results (Buck et al., 2011; Rosen et al., 2007). PFAS encompass a large family of over 15,000 chemicals (Sunderland et al., 2019). Their biological activity depends heavily on factors like carbon chain length and functional group (ITRC, 2023). For instance, activation of mouse and human PPARα by perfluoroalkyl carboxylates shows a positive correlation with carbon chain length (up to C9), and carboxylates tend to be stronger activators than sulfonates (Wolf et al., 2008). The move toward replacement PFAS (e.g., GenX) adds complexity, as these compounds may have distinct toxicological profiles or can rely on different molecular pathways compared to legacy compounds like PFOA (Attema et al., 2022; ITRC, 2023).

Interventional studies aiming to mitigate PFAS toxicity have yielded mixed results, highlighting the need for more targeted and effective therapeutic strategies. For example, some dietary interventions, specifically soluble fibers like inulin and pectin, have shown promise in reducing PFOS-induced liver metabolic disturbances and accumulation in mouse models (Deng et al., 2022). This protective effect is linked to the fiber’s ability to modulate transporters that reduce PFOS reabsorption (Deng et al., 2022). Similarly, vitamin C supplementation has been shown to reduce signs of PFOA-induced hepatotoxicity in mice (Li R. et al., 2022). However, generalized clinical guidance still focuses primarily on blood testing and regular screenings for high-risk individuals, reflecting the lack of established, proven therapeutic interventions for PFAS toxicity in humans (National Academies of Sciences Engineering and Medicine, 2022).

Future research should prioritize longitudinal human studies that include detailed lipidomic and epigenomic profiling to clarify links between PFAS exposure, metabolic disruption, and cancer risk. There should be investigations into emerging PFAS compounds and their new biological effects as legacy PFAS (PFOS and PFOA) are phased out. In addition, there should be more research into novel pharmacological or dietary interventions targeting PFAS-induced dysregulation of lipid metabolism and associated signaling pathways. This would have potential for reducing PFAS-related carcinogenic risk. These comprehensive efforts would be essential to inform public health policies and develop effective mitigation strategies against the growing global burden of PFAS contamination.

## References

[B1] Acharya R. Shetty S. S. Kumari N. S. (2023). Fatty acid transport proteins (FATPs) in cancer. Chem. Phys. Lipids 250, 105269. 10.1016/j.chemphyslip.2022.105269 36462545

[B2] Agency for Toxic Substances and Disease Registry (2021). Toxicological profile for perfluoroalkyls. Atlanta, GA: Centers for Disease Control and Prevention, 762. 37220203

[B3] Alijagic A. Sinisalu L. Duberg D. Kotlyar O. Scherbak N. Engwall M. (2024). Metabolic and phenotypic changes induced by PFAS exposure in two human hepatocyte cell models. Environ. Int. 190, 108820. 10.1016/j.envint.2024.108820 38906088

[B4] Attema B. Janssen A. W. F. Rijkers D. van Schothorst E. M. Hooiveld G. Kersten S. (2022). Exposure to low-dose perfluorooctanoic acid promotes hepatic steatosis and disrupts the hepatic transcriptome in mice. Mol. Metab. 66, 101602. 10.1016/j.molmet.2022.101602 36115532 PMC9526138

[B5] Bagley B. D. Chang S. C. Ehresman D. J. Eveland A. Zitzow J. D. Parker G. A. (2017). Perfluorooctane sulfonate-induced hepatic steatosis in Male sprague dawley rats is not attenuated by dietary choline supplementation. Toxicol. Sci. 160, 284–298. 10.1093/toxsci/kfx185 28973659

[B6] Barry V. Winquist A. Steenland K. (2013). Perfluorooctanoic acid (PFOA) exposures and incident cancers among adults living near a chemical plant. Environ. Health Persp 121, 1313–1318. 10.1289/ehp.1306615 24007715 PMC3855514

[B7] Beggs K. M. McGreal S. R. McCarthy A. Gunewardena S. Lampe J. N. Lau C. (2016). The role of hepatocyte nuclear factor 4-alpha in perfluorooctanoic acid- and perfluorooctanesulfonic acid-induced hepatocellular dysfunction. Toxicol. Appl. Pharmacol. 304, 18–29. 10.1016/j.taap.2016.05.001 27153767 PMC5367386

[B8] Behr A. C. Lichtenstein D. Braeuning A. Lampen A. Buhrke T. (2018). Perfluoroalkylated substances (PFAS) affect neither estrogen and androgen receptor activity nor steroidogenesis in human cells *in vitro* . Toxicol. Lett. 291, 51–60. 10.1016/j.toxlet.2018.03.029 29601859

[B9] Behr A. C. Plinsch C. Braeuning A. Buhrke T. (2020). Activation of human nuclear receptors by perfluoroalkylated substances (PFAS). Toxicol Vitro 62, 104700. 10.1016/j.tiv.2019.104700 31676336

[B10] Beloribi-Djefaflia S. Vasseur S. Guillaumond F. (2016). Lipid metabolic reprogramming in cancer cells. Oncogenesis 5, e189. 10.1038/oncsis.2015.49 26807644 PMC4728678

[B11] Berger J. Moller D. E. (2002). The mechanisms of action of PPARs. Annu. Rev. Med. 53, 409–435. 10.1146/annurev.med.53.082901.104018 11818483

[B12] Bian X. Liu R. Meng Y. Xing D. Xu D. Lu Z. (2021). Lipid metabolism cancer. J. Exp. Med. 218, e20201606. 10.1084/jem.20201606 33601415 PMC7754673

[B13] Bintee B. Banerjee R. Hegde M. Vishwa R. Alqahtani M. S. Abbas M. (2025). Exploring bile acid transporters as key players in cancer development and treatment: evidence from preclinical and clinical studies. Cancer Lett. 609, 217324. 10.1016/j.canlet.2024.217324 39571783

[B14] Birchfield A. S. Musayev F. N. Castillo A. J. Zorn G. Fuglestad B. (2025). Broad PFAS binding with fatty acid binding protein 4 is enabled by variable binding modes. bioRxiv. 10.1021/jacsau.5c00504 40575325 PMC12188406

[B15] Bjork J. A. Butenhoff J. L. Wallace K. B. (2011). Multiplicity of nuclear receptor activation by PFOA and PFOS in primary human and rodent hepatocytes. Toxicology 288, 8–17. 10.1016/j.tox.2011.06.012 21723365

[B16] Boyd R. I. Ahmad S. Singh R. Fazal Z. Prins G. S. Erdogan Z. M. (2022). Toward a mechanistic understanding of Poly- and perfluoroalkylated substances and cancer. Cancers 14, 2919. 10.3390/cancers14122919 35740585 PMC9220899

[B17] Boyd R. I. Shokry D. Fazal Z. Rennels B. C. Freemantle S. J. La Frano M. R. (2024). Perfluorooctanesulfonic acid alters pro-cancer phenotypes and metabolic and transcriptional signatures in testicular germ cell tumors. Toxics 12, 232. 10.3390/toxics12040232 38668455 PMC11054796

[B18] Boyd R. I. Shokry D. Rennels B. C. Adam Y. Powell C. Johnson S. (2025). The role of lipid metabolism and peroxisome proliferator activation in mediating pro-cancer phenotypes of poly- and perfluoroalkyl substances in testicular cancer. Environ. Toxicol. Pharmacol. 120, 104866. 10.1016/j.etap.2025.104866 41237835 PMC13371115

[B19] Broadfield L. A. Pane A. A. Talebi A. Swinnen J. V. Fendt S. M. (2021). Lipid metabolism in cancer: new perspectives and emerging mechanisms. Dev. Cell 56, 1363–1393. 10.1016/j.devcel.2021.04.013 33945792

[B20] Buck R. C. Franklin J. Berger U. Conder J. M. Cousins I. T. De Voogt P. (2011). Perfluoroalkyl and polyfluoroalkyl substances in the environment: terminology, classification, and origins. Integr. Environmental Assessment Management 7, 513–541. 10.1002/ieam.258 21793199 PMC3214619

[B21] Cao H. Zhou Z. Hu Z. Wei C. Li J. Wang L. (2022). Effect of enterohepatic circulation on the accumulation of Per- and polyfluoroalkyl substances: evidence from experimental and computational studies. Environ. Sci. Technol. 56, 3214–3224. 10.1021/acs.est.1c07176 35138827

[B22] Chen M. Huang J. T. (2019). The expanded role of fatty acid metabolism in cancer: new aspects and targets. Precis. Clin. Med. 2, 183–191. 10.1093/pcmedi/pbz017 31598388 PMC6770278

[B23] Cheng C. Geng F. Cheng X. Guo D. (2018). Lipid metabolism reprogramming and its potential targets in cancer. Cancer Commun. (Lond) 38, 27. 10.1186/s40880-018-0301-4 29784041 PMC5993136

[B24] Cheng H. Wang M. Su J. Li Y. Long J. Chu J. (2022). Lipid metabolism and cancer. Life (Basel) 12, 784. 10.3390/life12060784 35743814 PMC9224822

[B25] Currie E. Schulze A. Zechner R. Walther T. C. Farese R. V. Jr. (2013). Cellular fatty acid metabolism and cancer. Cell Metab. 18, 153–161. 10.1016/j.cmet.2013.05.017 23791484 PMC3742569

[B26] Das K. P. Wood C. R. Lin M. T. Starkov A. A. Lau C. Wallace K. B. (2017). Perfluoroalkyl acids-induced liver steatosis: effects on genes controlling lipid homeostasis. Toxicology 378, 37–52. 10.1016/j.tox.2016.12.007 28049043 PMC5994610

[B27] DeBerardinis R. J. Lum J. J. Hatzivassiliou G. Thompson C. B. (2008). The biology of cancer: metabolic reprogramming fuels cell growth and proliferation. Cell Metab. 7, 11–20. 10.1016/j.cmet.2007.10.002 18177721

[B28] Deng P. Durham J. Liu J. Zhang X. Wang C. Li D. (2022). Metabolomic, lipidomic, transcriptomic, and metagenomic analyses in mice exposed to PFOS and fed soluble and insoluble dietary fibers. Environ. Health Perspect. 130, 117003. 10.1289/EHP11360 36331819 PMC9635512

[B29] Dunn F. Paquette S. E. Pennell K. D. Plavicki J. S. Manz K. E. (2024). Metabolomic changes following GenX and PFBS exposure in developing zebrafish. Aquat. Toxicol. 271, 106908. 10.1016/j.aquatox.2024.106908 38608566 PMC11209921

[B30] Durham J. Tessmann J. W. Deng P. Hennig B. Zaytseva Y. Y. (2023). The role of perfluorooctane sulfonic acid (PFOS) exposure in inflammation of intestinal tissues and intestinal carcinogenesis. Front. Toxicol. 5, 1244457. 10.3389/ftox.2023.1244457 37662676 PMC10469509

[B31] Elcombe C. R. Elcombe B. M. Foster J. R. Chang S. C. Ehresman D. J. Butenhoff J. L. (2012). Hepatocellular hypertrophy and cell proliferation in sprague-dawley rats from dietary exposure to potassium perfluorooctanesulfonate results from increased expression of xenosensor nuclear receptors PPARalpha and CAR/PXR. Toxicology 293, 16–29. 10.1016/j.tox.2011.12.014 22245121

[B32] Feng Y. Bai Y. Lu Y. Chen M. Fu M. Guan X. (2022). Plasma perfluoroalkyl substance exposure and incidence risk of breast cancer: a case-cohort study in the dongfeng-tongji cohort. Environ. Pollut. 306, 119345. 10.1016/j.envpol.2022.119345 35472559

[B33] Fleishman J. S. Kumar S. (2024). Bile acid metabolism and signaling in health and disease: molecular mechanisms and therapeutic targets. Signal Transduct. Target Ther. 9, 97. 10.1038/s41392-024-01811-6 38664391 PMC11045871

[B34] Frisbee S. J. Shankar A. Knox S. S. Steenland K. Savitz D. A. Fletcher T. (2010). Perfluorooctanoic acid, perfluorooctanesulfonate, and serum lipids in children and adolescents: results from the C8 health project. Arch. Pediatr. Adolesc. Med. 164, 860–869. 10.1001/archpediatrics.2010.163 20819969 PMC3116641

[B35] Fu Y. Zou T. T. Shen X. T. Nelson P. J. Li J. H. Wu C. (2021). Lipid metabolism in cancer progression and therapeutic strategies. Medcomm 2, 27–59. 10.1002/mco2.27 34766135 PMC8491217

[B36] Geng F. Guo D. (2024). SREBF1/SREBP-1 concurrently regulates lipid synthesis and lipophagy to maintain lipid homeostasis and tumor growth. Autophagy 20, 1183–1185. 10.1080/15548627.2023.2275501 37927089 PMC11135807

[B37] Geng F. Zhong Y. Su H. Lefai E. Magaki S. Cloughesy T. F. (2023). SREBP-1 upregulates lipophagy to maintain cholesterol homeostasis in brain tumor cells. Cell Rep. 42, 112790. 10.1016/j.celrep.2023.112790 37436895 PMC10528745

[B38] Girardi P. Merler E. (2019). A mortality study on Male subjects exposed to polyfluoroalkyl acids with high internal dose of perfluorooctanoic acid. Environ. Res. 179, 108743. 10.1016/j.envres.2019.108743 31542491

[B39] Gou X. Tian M. Yan L. Xia P. Ji H. Tan H. (2024). A novel molecular pathway of lipid accumulation in human hepatocytes caused by PFOA and PFOS. Environ. Int. 191, 108962. 10.1016/j.envint.2024.108962 39159514

[B40] Grandjean P. Clapp R. (2015). Perfluorinated alkyl substances: emerging insights into health risks. New Solut. 25, 147–163. 10.1177/1048291115590506 26084549 PMC6172956

[B41] Guo P. Furnary T. Vasiliou V. Yan Q. Nyhan K. Jones D. P. (2022). Non-targeted metabolomics and associations with per- and polyfluoroalkyl substances (PFAS) exposure in humans: a scoping review. Environ. Int. 162, 107159. 10.1016/j.envint.2022.107159 35231839 PMC8969205

[B42] Gupta A. Das D. Taneja R. (2024). Targeting dysregulated lipid metabolism in cancer with pharmacological inhibitors. Cancers 16, 1313. 10.3390/cancers16071313 38610991 PMC11010992

[B43] Hammarstrand S. Jakobsson K. Andersson E. Xu Y. Li Y. Olovsson M. (2021). Perfluoroalkyl substances (PFAS) in drinking water and risk for polycystic ovarian syndrome, uterine leiomyoma, and endometriosis: a Swedish cohort study. Environ. Int. 157, 106819. 10.1016/j.envint.2021.106819 34391986

[B44] Hanahan D. Weinberg R. A. (2011). Hallmarks of cancer: the next generation. Cell 144, 646–674. 10.1016/j.cell.2011.02.013 21376230

[B45] Hari A. AbdulHameed M. D. M. Balik-Meisner M. R. Mav D. Phadke D. P. Scholl E. H. (2024). Exposure to PFAS chemicals induces sex-dependent alterations in key rate-limiting steps of lipid metabolism in liver steatosis. Front. Toxicol. 6, 1390196. 10.3389/ftox.2024.1390196 38903859 PMC11188372

[B46] Haug M. Dunder L. Lind P. M. Lind L. Salihovic S. (2023). Associations of perfluoroalkyl substances (PFAS) with lipid and lipoprotein profiles. J. Expo. Sci. Environ. Epidemiol. 33, 757–765. 10.1038/s41370-023-00545-x 37019983 PMC10541331

[B47] Hocevar S. E. Kamendulis L. M. Hocevar B. A. (2020). Perfluorooctanoic acid activates the unfolded protein response in pancreatic acinar cells. J. Biochem. Mol. Toxicol. 34, e22561. 10.1002/jbt.22561 32578922 PMC7655521

[B48] Hong Y. G. Wang D. Q. Liu Z. Y. Chen Y. X. Wang Y. Li J. J. (2025). Decoding per- and polyfluoroalkyl substances (PFAS) in hepatocellular carcinoma: a multi-omics and computational toxicology approach. J. Transl. Med. 23, 504. 10.1186/s12967-025-06517-z 40317014 PMC12049027

[B49] Hu W. Y. Lu R. L. Hu D. P. Imir O. B. Zuo Q. Y. Moline D. (2022). Per- and polyfluoroalkyl substances target and alter human prostate stem-progenitor cells. Biochem. Pharmacol. 197, 114902. 10.1016/j.bcp.2021.114902 34968493 PMC8890783

[B50] Huang Q. Luo L. Han X. Li F. Zhang X. Tian M. (2022). Low-dose perfluorooctanoic acid stimulates steroid hormone synthesis in leydig cells: integrated proteomics and metabolomics evidence. J. Hazard Mater 424, 127656. 10.1016/j.jhazmat.2021.127656 34774353

[B51] Imir O. B. Kaminsky A. Z. Zuo Q. Y. Liu Y. J. Singh R. Spinella M. J. (2021). Per- and polyfluoroalkyl substance exposure combined with high-fat diet supports prostate cancer progression. Nutrients 13, 3902. 10.3390/nu13113902 34836157 PMC8623692

[B52] India-Aldana S. Yao M. Midya V. Colicino E. Chatzi L. Chu J. (2023). PFAS exposures and the human metabolome: a systematic review of epidemiological studies. Curr. Pollut. Rep. 9, 510–568. 10.1007/s40726-023-00269-4 37753190 PMC10520990

[B53] ITRC (2023). Per- and polyfluoroalkyl substances team, Per- and polyfluroalkyl sunstances (PFAS).

[B54] Jia P. Yu X. Jin Y. Wang X. Yang A. Zhang L. (2024). Relationship between per-fluoroalkyl and polyfluoroalkyl substance exposure and insulin resistance in nondiabetic adults: evidence from NHANES 2003-2018. Ecotoxicol. Environ. Saf. 287, 117260. 10.1016/j.ecoenv.2024.117260 39504878

[B55] Kamendulis L. M. Wu Q. Sandusky G. E. Hocevar B. A. (2014). Perfluorooctanoic acid exposure triggers oxidative stress in the mouse pancreas. Toxicol. Rep. 1, 513–521. 10.1016/j.toxrep.2014.07.015 28962265 PMC5598264

[B56] Kashobwe L. Sadrabadi F. Brunken L. Coelho A. Sandanger T. M. Braeuning A. (2024a). Legacy and alternative per- and polyfluoroalkyl substances (PFAS) alter the lipid profile of HepaRG cells. Toxicology 506, 153862. 10.1016/j.tox.2024.153862 38866127

[B57] Kashobwe L. Sadrabadi F. Braeuning A. Leonards P. E. G. Buhrke T. Hamers T. (2024b). *In vitro* screening of understudied PFAS with a focus on lipid metabolism disruption. Archives Toxicol. 98, 3381–3395. 10.1007/s00204-024-03814-2 38953992 PMC11402862

[B58] Kelson C. O. Tessmann J. W. Geisen M. E. He D. Wang C. Gao T. (2024). Upregulation of fatty acid synthase increases activity of beta-catenin and expression of NOTUM to enhance stem-like properties of colorectal cancer cells. Cells 13, 1663. 10.3390/cells13191663 39404424 PMC11475157

[B59] Kirkwood-Donelson K. I. Chappel J. Tobin E. Dodds J. N. Reif D. M. DeWitt J. C. (2024). Investigating mouse hepatic lipidome dysregulation following exposure to emerging per- and polyfluoroalkyl substances (PFAS). Chemosphere 354, 141654. 10.1016/j.chemosphere.2024.141654 38462188 PMC10995748

[B60] Lai K. P. Li J. W. Cheung A. Li R. Billah M. B. Chan T. F. (2017). Transcriptome sequencing reveals prenatal PFOS exposure on liver disorders. Environ. Pollut. 223, 416–425. 10.1016/j.envpol.2017.01.041 28131474

[B61] Lee Y. Y. Wong C. K. Oger C. Durand T. Galano J. M. Lee J. C. (2015). Prenatal exposure to the contaminant perfluorooctane sulfonate elevates lipid peroxidation during mouse fetal development but not in the pregnant dam. Free Radic. Res. 49, 1015–1025. 10.3109/10715762.2015.1027199 25787935

[B62] Lee W. K. Lam T. K. Y. Tang H. C. Ho T. C. Wan H. T. Wong C. K. C. (2023). PFOS-Elicited metabolic perturbation in liver and fatty acid metabolites in testis of adult mice. Front. Endocrinol. (Lausanne) 14, 1302965. 10.3389/fendo.2023.1302965 38075064 PMC10703039

[B63] Li X. Wang Z. Klaunig J. E. (2019). The effects of perfluorooctanoate on high fat diet induced non-alcoholic fatty liver disease in mice. Toxicology 416, 1–14. 10.1016/j.tox.2019.01.017 30711707

[B64] Li R. Guo C. Lin X. Chan T. F. Lai K. P. Chen J. (2020). Integrative omics analyses uncover the mechanism underlying the immunotoxicity of perfluorooctanesulfonate in human lymphocytes. Chemosphere 256, 127062. 10.1016/j.chemosphere.2020.127062 32434090

[B65] Li H. Hammarstrand S. Midberg B. Xu Y. Li Y. Olsson D. S. (2022a). Cancer incidence in a Swedish cohort with high exposure to perfluoroalkyl substances in drinking water. Environ. Res. 204, 112217. 10.1016/j.envres.2021.112217 34662573

[B66] Li R. Guo C. Lin X. Chan T. F. Su M. Zhang Z. (2022b). Integrative omics analysis reveals the protective role of vitamin C on perfluorooctanoic acid-induced hepatoxicity. J. Adv. Res. 35, 279–294. 10.1016/j.jare.2021.04.003 35024202 PMC8721266

[B67] Li R. Zhang Z. Xuan Y. Wang Y. Zhong Y. Zhang L. (2024). HNF4A as a potential target of PFOA and PFOS leading to hepatic steatosis: integrated molecular docking, molecular dynamic and transcriptomic analyses. Chem. Biol. Interact. 390, 110867. 10.1016/j.cbi.2024.110867 38199259

[B68] Li S. Q. Yuan H. Li L. Li Q. Lin P. Li K. (2025a). Oxidative stress and reprogramming of lipid metabolism in cancers. Antioxidants-Basel 14, 201. 10.3390/antiox14020201 40002387 PMC11851681

[B69] Li X. Jing K. He L. Song P. Yu J. (2025b). Impact of per- and polyfluoroalkyl substances structure on oxidative stress and lipid metabolism disruption in HepG2 cells. Toxicology 517, 154218. 10.1016/j.tox.2025.154218 40516594

[B70] Lin C. Y. Lee H. L. Hwang Y. T. Su T. C. (2020). The association between total serum isomers of per- and polyfluoroalkyl substances, lipid profiles, and the DNA oxidative/nitrative stress biomarkers in middle-aged Taiwanese adults. Environ. Res. 182, 109064. 10.1016/j.envres.2019.109064 31884197

[B71] Liu Q. Liu Y. Li X. Wang D. Zhang A. Pang J. (2023). Perfluoroalkyl substances promote breast cancer progression *via* ERalpha and GPER mediated PI3K/Akt and MAPK/erk signaling pathways. Ecotoxicol. Environ. Saf. 258, 114980. 10.1016/j.ecoenv.2023.114980 37148752

[B72] Louisse J. Rijkers D. Stoopen G. Janssen A. Staats M. Hoogenboom R. (2020). Perfluorooctanoic acid (PFOA), perfluorooctane sulfonic acid (PFOS), and perfluorononanoic acid (PFNA) increase triglyceride levels and decrease cholesterogenic gene expression in human HepaRG liver cells. Arch. Toxicol. 94, 3137–3155. 10.1007/s00204-020-02808-0 32588087 PMC7415755

[B73] Maerten A. Callewaert E. Sanz-Serrano J. Devisscher L. Vinken M. (2024). Effects of per- and polyfluoroalkyl substances on the liver: human-Relevant mechanisms of toxicity. Sci. Total Environ. 954, 176717. 10.1016/j.scitotenv.2024.176717 39383969

[B74] Mancini F. R. Cano-Sancho G. Gambaretti J. Marchand P. Boutron-Ruault M. C. Severi G. (2020). Perfluorinated alkylated substances serum concentration and breast cancer risk: evidence from a nested case-control study in the French E3N cohort. Int. J. Cancer 146, 917–928. 10.1002/ijc.32357 31008526

[B75] Marques E. Pfohl M. Auclair A. Jamwal R. Barlock B. J. Sammoura F. M. (2020). Perfluorooctanesulfonic acid (PFOS) administration shifts the hepatic proteome and augments dietary outcomes related to hepatic steatosis in mice. Toxicol. Appl. Pharmacol. 408, 115250. 10.1016/j.taap.2020.115250 32979393 PMC8386191

[B76] Mei J. Jiang J. Li Z. Pan Y. Xu K. Gao X. (2024). Increased perfluorooctanoic acid accumulation facilitates the migration and invasion of lung cancer cells *via* remodeling cell mechanics. Proc. Natl. Acad. Sci. U. S. A. 121, e2408575121. 10.1073/pnas.2408575121 39665760 PMC11665856

[B77] Molendijk J. Robinson H. Djuric Z. Hill M. M. (2020). Lipid mechanisms in hallmarks of cancer. Mol. Omics 16, 6–18. 10.1039/c9mo00128j 31755509 PMC7184895

[B78] Mueller R. Schlosser K. E. (2020). History and use of Per- and polyfluoroalkyl substances (PFAS) found in the environment. Washington, DC.

[B79] National Academies of Sciences Engineering and Medicine (2022). Guidance on PFAS exposure, testing, and clinical Follow-Up. Washington, DC: The National Academies Press, 300. 35939564

[B80] NCfBI (2026a). PubChem compound summary for CID 9883, Hexafluoropropylene oxide. Available online at: https://pubchem.ncbi.nlm.nih.gov/compound/Hexafluoropropylene-oxide (Accessed February 16, 2026).

[B81] NCfBI (2026b). PubChem compound summary for CID 74483, Perfluorooctanesulfonic acid. Available onlie at: https://pubchem.ncbi.nlm.nih.gov/compound/Perfluorooctanesulfonic-acid (Accessed February 16, 2026).

[B82] NCfBI (2026c). PubChem compound summary for CID 67818, Perfluoroheptanoic acid.

[B83] NCfBI (2026d). PubChem compound summary for CID 67821, Perfluorononanoic acid. Available online at: https://pubchem.ncbi.nlm.nih.gov/compound/Perfluorononanoic-acid (Accessed February 16, 2026).

[B84] NCfBI (2026e). PubChem compound summary for CID 67734, Perfluorohexanesulfonic Acid. PubChem. Available online at: https://pubchem.ncbi.nlm.nih.gov/compound/Perfluorohexanesulfonic-acid (Accessed February 16, 2026).

[B85] NCfBI (2026f). PubChem compound summary for CID 9555, Perfluorodecanoic acid.

[B86] NCfBI (2026g). PubChem compound summary for CID 3816071, lithium bis((trifluoromethyl)sulfonyl)azanide.

[B137] NCfBI (2026h). PubChem compound summary for CID 9554, Perfluorooctanoic acid. Available online at: https://pubchem.ncbi.nlm.nih.gov/compound/Perfluorooctanoic-acid (Accessed February 16, 2026).

[B87] Nian M. Luo K. Luo F. Aimuzi R. Huo X. Chen Q. (2020). Association between prenatal exposure to PFAS and fetal sex hormones: are the short-chain PFAS safer? Environ. Sci. and Technol. 54, 8291–8299. 10.1021/acs.est.0c02444 32525661

[B88] Ouidir M. Mendola P. Buck Louis G. M. Kannan K. Zhang C. Tekola-Ayele F. (2020). Concentrations of persistent organic pollutants in maternal plasma and epigenome-wide placental DNA methylation. Clin. Epigenetics 12, 103. 10.1186/s13148-020-00894-6 32653021 PMC7371466

[B89] Paddayuman J. Z. Cristobal J. R. Landau L. J. B. Gagnon A. L. Gokcumen O. Aga D. S. (2025). Chronic exposure to PFAS triggers systems-level cellular reprogramming independent of their bioaccumulation. bioRxiv. 10.1101/2025.07.03.662990 40672246 PMC12265641

[B90] Pang W. K. Kuznetsova E. Holota H. De Haze A. Beaudoin C. Volle D. H. (2024). Understanding the role of endocrine disrupting chemicals in testicular germ cell cancer: insights into molecular mechanisms. Mol. Asp. Med. 99, 101307. 10.1016/j.mam.2024.101307 39213722

[B91] Pascual G. Avgustinova A. Mejetta S. Martin M. Castellanos A. Attolini C. S. (2017). Targeting metastasis-initiating cells through the fatty acid receptor CD36. Nature 541, 41–45. 10.1038/nature20791 27974793

[B92] Pierozan P. Cattani D. Karlsson O. (2020). Perfluorooctane sulfonate (PFOS) and perfluorooctanoic acid (PFOA) induce epigenetic alterations and promote human breast cell carcinogenesis *in vitro* . Arch. Toxicol. 94, 3893–3906. 10.1007/s00204-020-02848-6 32700164 PMC7603464

[B93] Prentice K. J. Saksi J. Robertson L. T. Lee G. Y. Inouye K. E. Eguchi K. (2021). A hormone complex of FABP4 and nucleoside kinases regulates islet function. Nature 600, 720–726. 10.1038/s41586-021-04137-3 34880500 PMC8983123

[B95] Purdue M. P. Rhee J. Denic-Roberts H. McGlynn K. A. Byrne C. Sampson J. (2023). A nested case-control Study of serum Per- and Polyfluoroalkyl substances and testicular germ cell tumors among U.S. air force servicemen. Environ. Health Perspect. 131, 77007. 10.1289/EHP12603 37458713 PMC10351502

[B96] Qi Q. Niture S. Gadi S. Arthur E. Moore J. Levine K. E. (2023). Per- and polyfluoroalkyl substances activate UPR pathway, induce steatosis and fibrosis in liver cells. Environ. Toxicol. 38, 225–242. 10.1002/tox.23680 36251517 PMC10092267

[B97] Rosen M. B. Thibodeaux J. R. Wood C. R. Zehr R. D. Schmid J. E. Lau C. (2007). Gene expression profiling in the lung and liver of PFOA-exposed mouse fetuses. Toxicology 239, 15–33. 10.1016/j.tox.2007.06.095 17681415

[B98] Rosen M. B. Schmid J. R. Corton J. C. Zehr R. D. Das K. P. Abbott B. D. (2010). Gene expression profiling in wild-type and PPARalpha-Null mice exposed to perfluorooctane sulfonate reveals PPARalpha-Independent effects. PPAR Res. 2010. 10.1155/2010/794739 20936131 PMC2948942

[B99] Rosen M. B. Das K. P. Wood C. R. Wolf C. J. Abbott B. D. Lau C. (2013). Evaluation of perfluoroalkyl acid activity using primary mouse and human hepatocytes. Toxicology 308, 129–137. 10.1016/j.tox.2013.03.011 23567314

[B100] Rosen M. B. Das K. P. Rooney J. Abbott B. Lau C. Corton J. C. (2017). PPARalpha-independent transcriptional targets of perfluoroalkyl acids revealed by transcript profiling. Toxicology 387, 95–107. 10.1016/j.tox.2017.05.013 28558994 PMC6129013

[B101] Rosen E. M. Kotlarz N. Knappe D. R. U. Lea C. S. Collier D. N. Richardson D. B. (2022). Drinking water-associated PFAS and fluoroethers and lipid outcomes in the GenX exposure Study. Environ. Health Perspect. 130, 97002. 10.1289/EHP11033 36069575 PMC9450637

[B102] Roth K. Yang Z. Agarwal M. Liu W. Peng Z. Long Z. (2021). Exposure to a mixture of legacy, alternative, and replacement per- and polyfluoroalkyl substances (PFAS) results in sex-dependent modulation of cholesterol metabolism and liver injury. Environ. Int. 157, 106843. 10.1016/j.envint.2021.106843 34479135 PMC8490327

[B103] Roth K. Yang Z. Agarwal M. Birbeck J. Westrick J. Lydic T. (2024). Exposure of Ldlr-/- mice to a PFAS mixture and outcomes related to circulating lipids, bile acid excretion, and the Intestinal Transporter ASBT. Environ. Health Perspect. 132, 87007. 10.1289/EHP14339 39177951 PMC11343043

[B104] Sadrabadi F. Alarcan J. Sprenger H. Braeuning A. Buhrke T. (2024). Impact of perfluoroalkyl substances (PFAS) and PFAS mixtures on lipid metabolism in differentiated HepaRG cells as a model for human hepatocytes. Arch. Toxicol. 98, 507–524. 10.1007/s00204-023-03649-3 38117326 PMC10794458

[B105] Sands M. Zhang X. Gal A. Laws M. Spinella M. Erdogan Z. M. (2024). Comparative hepatotoxicity of novel lithium bis(trifluoromethanesulfonyl)imide (LiTFSI, ie. HQ-115) and legacy Perfluorooctanoic acid (PFOA) in male mice: insights into epigenetic mechanisms and pathway-specific responses. Environ. Int. 185, 108556. 10.1016/j.envint.2024.108556 38461777 PMC12233031

[B106] Sang L. Wang X. Bai W. Shen J. Zeng Y. Sun J. (2022). The role of hepatocyte nuclear factor 4alpha (HNF4alpha) in tumorigenesis. Front. Oncol. 12, 1011230. 10.3389/fonc.2022.1011230 36249028 PMC9554155

[B107] Scharmach E. Buhrke T. Lichtenstein D. Lampen A. (2012). Perfluorooctanoic acid affects the activity of the hepatocyte nuclear factor 4 alpha (HNF4alpha). Toxicol. Lett. 212, 106–112. 10.1016/j.toxlet.2012.05.007 22609092

[B108] Sheng N. Cui R. Wang J. Guo Y. Wang J. Dai J. (2018). Cytotoxicity of novel fluorinated alternatives to long-chain perfluoroalkyl substances to human liver cell line and their binding capacity to human liver fatty acid binding protein. Arch. Toxicol. 92, 359–369. 10.1007/s00204-017-2055-1 28864880

[B109] Shi W. S. Zhang Z. L. Li X. Y. Chen J. S. Liang X. J. Li J. F. (2023). GenX disturbs the indicators of hepatic lipid metabolism Even at environmental concentration in drinking water *via* PPARα signaling pathways. Chem. Res. Toxicol. 37, 98–108. 10.1021/acs.chemrestox.3c00342 38150050

[B110] Sim K. H. Oh H. S. Lee C. Eun H. Lee Y. J. (2023). Evaluation of the effect of perfluorohexane sulfonate on the proliferation of human liver cells. Int. J. Environ. Res. Public Health 20, 6868. 10.3390/ijerph20196868 37835138 PMC10572997

[B111] Steenland K. Zhao L. Winquist A. (2015). A cohort incidence study of workers exposed to perfluorooctanoic acid (PFOA). Occup. Environ. Med. 72, 373–380. 10.1136/oemed-2014-102364 25601914

[B112] Steenland K. Tinker S. Frisbee S. Ducatman A. Vaccarino V. (2009). Association of perfluorooctanoic acid and perfluorooctane sulfonate with serum lipids among adults living near a chemical plant. Am. J. Epidemiol. 170, 1268–1278. 10.1093/aje/kwp279 19846564

[B113] Sunderland E. M. Hu X. C. Dassuncao C. Tokranov A. K. Wagner C. C. Allen J. G. (2019). A review of the pathways of human exposure to poly- and perfluoroalkyl substances (PFASs) and present understanding of health effects. J. Expo. Sci. Environ. Epidemiol. 29, 131–147. 10.1038/s41370-018-0094-1 30470793 PMC6380916

[B114] Tessmann J. W. Deng P. Durham J. Li C. Banerjee M. Wang Q. (2024). Perfluorooctanesulfonic acid exposure leads to downregulation of 3-hydroxy-3-methylglutaryl-CoA synthase 2 expression and upregulation of markers associated with intestinal carcinogenesis in mouse intestinal tissues. Chemosphere 359, 142332. 10.1016/j.chemosphere.2024.142332 38754493 PMC11157449

[B115] Vanden Heuvel J. P. Thompson J. T. Frame S. R. Gillies P. J. (2006). Differential activation of nuclear receptors by perfluorinated fatty acid analogs and natural fatty acids: a comparison of human, mouse, and rat peroxisome proliferator-activated receptor-alpha, -beta, and -gamma, liver X receptor-beta, and retinoid X receptor-alpha. Toxicol. Sci. 92, 476–489. 10.1093/toxsci/kfl014 16731579

[B116] Vieira V. M. Hoffman K. Shin H. M. Weinberg J. M. Webster T. F. Fletcher T. (2013). Perfluorooctanoic acid exposure and cancer outcomes in a contaminated community: a geographic analysis. Environ. Health Perspect. 121, 318–323. 10.1289/ehp.1205829 23308854 PMC3621179

[B117] Wan H. T. Zhao Y. G. Wei X. Hui K. Y. Giesy J. P. Wong C. K. (2012). PFOS-induced hepatic steatosis, the mechanistic actions on beta-oxidation and lipid transport. Biochim. Biophys. Acta 1820, 1092–1101. 10.1016/j.bbagen.2012.03.010 22484034

[B118] Wang L. Wang Y. Liang Y. Li J. Liu Y. Zhang J. (2013). Specific accumulation of lipid droplets in hepatocyte nuclei of PFOA-exposed BALB/c mice. Sci. Rep. 3, 2174. 10.1038/srep02174 23846197 PMC3709163

[B119] Wang X. Liu L. Zhang W. Zhang J. Du X. Huang Q. (2017). Serum metabolome biomarkers associate low-level environmental perfluorinated compound exposure with oxidative/nitrosative stress in humans. Environ. Pollut. 229, 168–176. 10.1016/j.envpol.2017.04.086 28599201

[B120] Wang L. Q. Liu T. Yang S. Sun L. Zhao Z. Y. Li L. Y. (2021). Perfluoroalkyl substance pollutants activate the innate immune system through the AIM2 inflammasome. Nat. Commun. 12, 2915. 10.1038/s41467-021-23201-0 34006824 PMC8131593

[B121] Wang T. Yang J. Han Y. Wang Y. (2024). Unveiling the intricate connection between per- and polyfluoroalkyl substances and prostate hyperplasia. Sci. Total Environ. 932, 173085. 10.1016/j.scitotenv.2024.173085 38729377

[B122] Wei R. Zhou Y. Li C. Rychahou P. Zhang S. Titlow W. B. (2022). Ketogenesis Attenuates KLF5-Dependent production of CXCL12 to overcome the immunosuppressive Tumor microenvironment in colorectal cancer. Cancer Res. 82, 1575–1588. 10.1158/0008-5472.CAN-21-2778 35247887 PMC9018557

[B123] Wen Y. Mirji N. Irudayaraj J. (2020). Epigenetic toxicity of PFOA and GenX in HepG2 cells and their role in lipid metabolism. Toxicol Vitro 65, 104797. 10.1016/j.tiv.2020.104797 32068100

[B124] Winquist A. Hodge J. M. Diver W. R. Rodriguez J. L. Troeschel A. N. Daniel J. (2023). Case-Cohort Study of the Association between PFAS and selected cancers among participants in the American cancer society's cancer prevention Study II LifeLink cohort. Environ. Health Perspect. 131, 127007. 10.1289/EHP13174 38088576 PMC10718084

[B125] Wolf C. J. Takacs M. L. Schmid J. E. Lau C. Abbott B. D. (2008). Activation of mouse and human peroxisome proliferator-activated receptor alpha by perfluoroalkyl acids of different functional groups and chain lengths. Toxicol. Sci. 106, 162–171. 10.1093/toxsci/kfn166 18713766

[B126] Wu Z. Ouyang T. Liu H. Cao L. Chen W. (2023). Perfluoroalkyl substance (PFAS) exposure and risk of nonalcoholic fatty liver disease in the elderly: results from NHANES 2003-2014. Environ. Sci. Pollut. Res. Int. 30, 64342–64351. 10.1007/s11356-023-26941-2 37067713

[B127] Yan S. Wang J. Dai J. (2015). Activation of sterol regulatory element-binding proteins in mice exposed to perfluorooctanoic acid for 28 days. Arch. Toxicol. 89, 1569–1578. 10.1007/s00204-014-1322-7 25092180

[B128] Yang D. Han J. Hall D. R. Sun J. Fu J. Kutarna S. (2020). Nontarget screening of Per- and Polyfluoroalkyl substances binding to human liver Fatty acid binding protein. Environ. Sci. Technol. 54, 5676–5686. 10.1021/acs.est.0c00049 32249562 PMC7477755

[B129] Yang W. Ling X. He S. Cui H. Yang Z. An H. (2023a). PPARalpha/ACOX1 as a novel target for hepatic lipid metabolism disorders induced by per- and polyfluoroalkyl substances: an integrated approach. Environ. Int. 178, 108138. 10.1016/j.envint.2023.108138 37572494

[B130] Yang Z. Y. Fu L. Cao M. X. Li F. Li J. G. Chen Z. Y. (2023b). PFAS-induced lipidomic dysregulations and their associations with developmental toxicity in zebrafish embryos. Sci. Total Environ. 861, 160691. 10.1016/j.scitotenv.2022.160691 36473658

[B131] Yu G. Wang J. Liu Y. Luo T. Meng X. Zhang R. (2023). Metabolic perturbations in pregnant rats exposed to low-dose perfluorooctanesulfonic acid: an integrated multi-omics analysis. Environ. Int. 173, 107851. 10.1016/j.envint.2023.107851 36863164

[B132] Zaytseva Y. (2021). Lipid metabolism as a targetable metabolic vulnerability in colorectal cancer. Cancers (Basel) 13, 301. 10.3390/cancers13020301 33467532 PMC7830794

[B133] Zhang J. J. Chen Y. K. Chen Y. Q. Zhang Q. Y. Liu Y. Wang Q. (2025). Gestational GenX exposure induces maternal hepatotoxicity by disrupting the lipid and bile acid metabolism distinguished from PFOA-Induced pyroptosis. Toxics 13, 617. 10.3390/toxics13080617 40863893 PMC12389976

[B134] Zhao W. Zitzow J. D. Ehresman D. J. Chang S. C. Butenhoff J. L. Forster J. (2015). Na+/Taurocholate cotransporting polypeptide and apical sodium-dependent bile acid transporter are involved in the disposition of Perfluoroalkyl sulfonates in humans and rats. Toxicol. Sci. 146, 363–373. 10.1093/toxsci/kfv102 26001962 PMC4607751

[B135] Zhao J. Zhi Z. Wang C. Xing H. Song G. Yu X. (2017). Exogenous lipids promote the growth of breast cancer cells *via* CD36. Oncol. Rep. 38, 2105–2115. 10.3892/or.2017.5864 28765876 PMC5652970

[B136] Zheng J. Liu S. Yang J. Zheng S. Sun B. (2024). Per- and polyfluoroalkyl substances (PFAS) and cancer: detection methodologies, epidemiological insights, potential carcinogenic mechanisms, and future perspectives. Sci. Total Environ. 953, 176158. 10.1016/j.scitotenv.2024.176158 39255941

