# Correction: Impact of PFAS exposure on lipid metabolic pathways: mechanisms and implications in carcinogenesis

**DOI:** 10.3389/ftox.2026.1832497

**Published:** 2026-03-25

**Authors:** Frontiers Production Office

**Affiliations:** Frontiers Media SA, Lausanne, Switzerland

**Keywords:** cancer, carcinogenesis, forever chemicals, lipid metabolism, PFAS exposure

The title of this article was corrected from “*Impact of PFAS exposure on lipid metabolic pathways: mechanisms and implicatins in carcinogenesis*” to “*Impact of PFAS exposure on lipid metabolic pathways: mechanisms and implications in carcinogenesis*”.

The original version of this article has been updated.

